# Aptamer-Based Targeting of Cancer: A Powerful Tool for Diagnostic and Therapeutic Aims

**DOI:** 10.3390/bios14020078

**Published:** 2024-01-31

**Authors:** Arash Mohammadinejad, Laura Elena Gaman, Ghazaleh Aleyaghoob, Liviu Gaceu, Seyed Ahmad Mohajeri, Marius Alexandru Moga, Mihaela Badea

**Affiliations:** 1 Department of Fundamental, Prophylactic and Clinical Disciplines, Faculty of Medicine, Transilvania University of Brasov, 500019 Brașov, Romania; mihaela.badea@unitbv.ro; 2 Research Center for Fundamental Research and Prevention Strategies in Medicine, Research and Development Institute of Transilvania University of Brasov, 500484 Brașov, Romania; 3 Faculty of Medicine, University of Medicine and Pharmacy “Carol Davila”, 020021 Bucharest, Romania; laura.gaman@umfcd.ro; 4 Department of Medical Biotechnology and Nanotechnology, School of Medicine, Mashhad University of Medical Sciences, Mashhad 9177948564, Iran; gh_aleyaghoob@yahoo.com; 5 Department of Chemistry, Payame Noor University, Tehran 19395-4697, Iran; 6 Faculty of Food and Tourism, Transilvania University of Brasov, 500014 Brașov, Romania; gaceul@unitbv.ro; 7 Pharmaceutical Research Center, Pharmaceutical Technology Institute, Mashhad University of Medical Sciences, Mashhad 9177948954, Iran; mohajeria@mums.ac.ir; 8 Department of Pharmacodynamics and Toxicology, School of Pharmacy, Mashhad University of Medical Sciences, Mashhad 9177948954, Iran; 9 Department of Medical and Surgical Specialties, Faculty of Medicine, Transilvania University of Brasov, 500019 Brașov, Romania; mogas@unitbv.ro; 10 Centre for Applied Medicine and Intervention Strategies in Medical Practice, Research and Development Institute of Transilvania University of Brasov, 500484 Brașov, Romania

**Keywords:** aptamer, cancer, diagnosis, therapy, biosensor

## Abstract

Cancer is known as one of the most significant causes of death worldwide, and, in spite of novel therapeutic methods, continues to cause a considerable number of deaths. Targeted molecular diagnosis and therapy using aptamers with high affinity have become popular techniques for pathological angiogenesis and cancer therapy scientists. In this paper, several aptamer-based diagnostic and therapeutic techniques such as aptamer–nanomaterial conjugation, aptamer–drug conjugation (physically or covalently), and biosensors, which have been successfully designed for biomarkers, were critically reviewed. The results demonstrated that aptamers can potentially be incorporated with targeted delivery systems and biosensors for the detection of biomarkers expressed by cancer cells. Aptamer-based therapeutic and diagnostic methods, representing the main field of medical sciences, possess high potential for use in cancer therapy, pathological angiogenesis, and improvement of community health. The clinical use of aptamers is limited due to target impurities, inaccuracy in the systematic evolution of ligands via exponential enrichment (SELEX)stage process, and in vitro synthesis, making them unreliable and leading to lower selectivity for in vivo targets. Moreover, size, behavior, probable toxicity, low distribution, and the unpredictable behavior of nanomaterials in in vivo media make their usage in clinical assays critical. This review is helpful for the implementation of aptamer-based therapies which are effective and applicable for clinical use and the design of future studies.

## 1. Introduction

Aptamers are known as synthetic nucleic acid strands that can specifically bind to a wide range of targets such as proteins, cells, bacteria, toxins, DNA, miRNA, ions, and drug molecules with high affinity [1]. Aptamers were first introduced in 1990 by Larry Gold and his colleagues at Colorado University during the process of systematic evolution of ligands via exponential enrichment (SELEX). Briefly, this technique includes the introduction of a target into a library of random sequences of ssRNA or ssDNA (10^13^–10^16^), cleaning up the unbounded strands, and washing and reproduction of the bounded strands with Polymerase Chain Reaction (PCR) (6 to 15 times) [2]. 

Although antibodies and aptamers, which are known as chemical antibodies, play the same role in terms of specific binding to targets, some advantages of aptamers over antibodies make their use preferred for designing targeted delivery and biosensing platforms. The interesting features of aptamers are their specificity to molecules in cells and also monoclonal antibodies, easy penetration into tissue and cells, easy and inexpensive production, and smaller size [3,4,5,6]. 

**Scheme 1:**
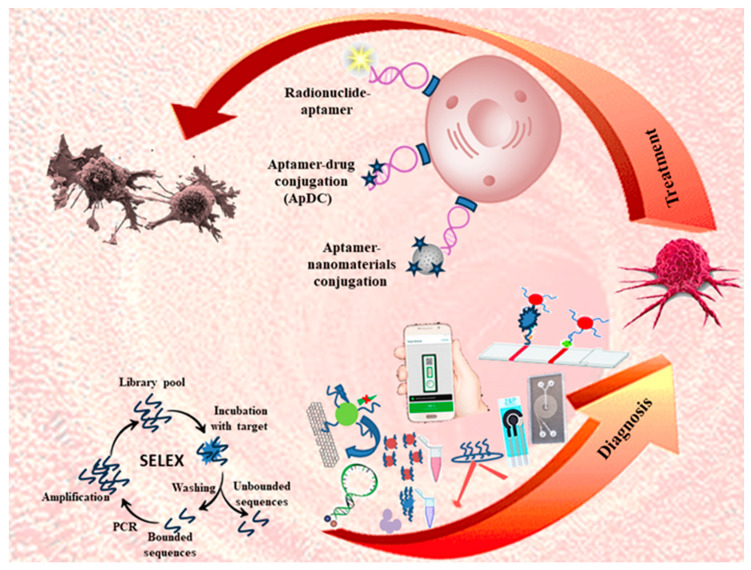
Application of aptamer as a therapeutic and diagnosis agent for tumors.

The folding of aptamers with secondary and tertiary conformation leads to high target affinity [1,7]. This feature enables the usage of aptamers as targeting and nanocarrier agents for the delivery of therapeutic and imaging agents to the tumor cell through the recognition of biomarkers. Moreover, aptamers can be used as therapeutic agents. According to the Food and Drug Administration (FDA), age-related macular degeneration can be treated with an aptamer-based drug, Macugen^®^ (pegaptanib) [8]. On the other hand, aptamers can be used for the detection of various biomarkers related to various diseases such as cardiovascular diseases, cancer, neurodegenerative diseases, infections, and organ damage. So, as shown in Scheme 1, aptamers can be implemented as powerful therapeutic and diagnosis agents. 

According to the American Cancer Society, about 1,918,030 cancer cases and 609,360 cancer deaths were estimated in 2022 in the United States. Moreover, about 350 deaths per day were recorded for lung cancer, which was the leading cause of death. However, the statistical studies of the National Center for Health Statistics (NCHS) of the USA demonstrated that the mortality patterns contain stagnated progression for prostate and breast cancers and an increase in lung cancer [9]. This statistical estimation shows the importance of cancer survival, which can be achieved by timely treatment and diagnosis. 

Biosensors with the ability to detect a wide range of biological molecules including DNA, RNA, and protein can play a role in the clinical process. In the clinical process, biosensors can be used for the detection of biomarkers or the evaluation of the effect of therapeutic agents at specific sites. Moreover, designing targeted delivery systems for monitoring, imaging, and therapeutic aims for various cancer cells and tumors plays a vital role in clinical and cancer therapy use. So, the incorporation of aptamers with the aforementioned platforms is important for the timely diagnosis and therapy of cancers [10]. 

Due to the importance of this subject, in this paper, we review the application of aptamers in designing targeted delivery systems and biosensors and critically discuss methods, which can be helpful for future studies.

## 2. Aptamer-Based Targeted Delivery Systems

Targeted delivery as a main field of medical sciences has been highly applied for cancer therapy and pathological angiogenesis. This process can be performed in the form of aptamer–nanomaterial conjugations and aptamer–drug conjugates (ApDCs). The former benefits from the high surface area of nanomaterials for loading the different anticancer drugs and possible therapeutic effects of nanomaterials like thermal or ROS production. The latter is mainly used for loading intercalating drugs such as doxorubicin (DOX), which reduces the toxicity of normal cells, by targeting specific cells [11,12]. The combination of an aptamer with therapeutic agents can resolve some problems such as low in vivo sensitivity and selectivity, the high toxicity of drugs, tumor imaging difficulty, etc. These systems can achieve cancer diagnosis or prognosis, and cancer signal pathways blocking [7]. In this section, the studies which employed the aptamer in targeted-delivery systems are discussed and their information is summarized in Table 1.

### 2.1. Aptamer–Nanomaterial Conjugation

Nanomaterials, as an inseparable part of targeted delivery systems, can be incorporated with aptamers for the aims of therapeutic and imaging systems. Nanomaterials are known as a favorable candidate for functionalization with aptamers due to the properties of biodegradability, high loading capacity, easy bioconjugation, high permeability, and easy release of drugs. Targeted delivery of drugs to cells and tissues can considerably reduce the cytotoxicity and enhance drug efficacy [34]. Moreover, the controllable release of drugs can happen because of the easy and fast response of nanoparticle carriers to the environmental condition or stimuli in cells [35]. This kind of targeted delivery synergically benefits from the advantages of both parts, including the high surface area of nanomaterials for the binding of aptamers, the protected situation of the aptamer from nuclease degradation, and high sensitivity and selectivity [36,37]. Here, we will discuss more on the nanoparticles which have been frequently used for designing the drug delivery systems such as gold nanoparticles (AuNPs) [38], carbon-based nanoparticles [39], quantum dots (QDs) [40,41], silica nanoparticles [42], polymeric nanomaterials [43], liposomes [44], magnetic nanoparticles [45], metal–organic frameworks (MOFs), and upconversion nanoparticles (UCNPs) [46]. 

**Figure 1:**
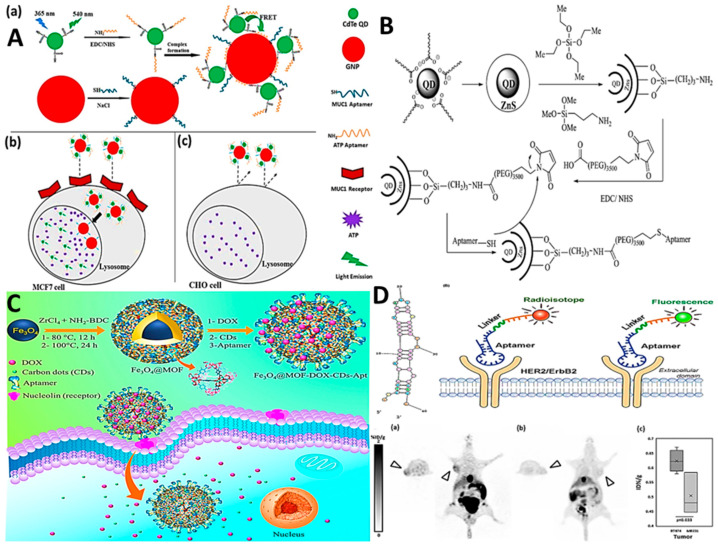
(**A**) Application of AuNPs and QDs in MCF-7 cell imaging: (**a**) preparation of MUC1 apt-AuNPs/ATP apt-QDs complex; (**b**) selective internalization of the complex into MCF7 cell by interaction between MUC1 aptamer and MUC1 biomarker overexpressed on the MCF7 cell surface. (**c**) Failure to enter of complex into CHO cell due to the absence of MUC1 biomarker (AuNP: GNP). “Reprinted from with [13] permission from Elsevier”. (**B**) Synthesis of Gd-Zn-Cu-In-S/ZnS QDs/PEG/EpCAM DNA and conjugation of thiolated apatamer with maleimide group. “Reprinted from [15] with permission from Elsevier”. (**C**) Synthesis of Fe_3_O_4_@ UiO-66-NH_2_ MOF-DOX-CDs-Aps aptamer nanocarrier and selective entrance to MDA-MB-231 human breast cancer cells through interaction of AS1411 aptamer with nucleolin biomarker. “Reprinted from [21] with permission from Elsevier”. (**D**) Application of ^18^F-fluoride- HER2 aptamer. Top: mechanism of the entrance to tumor cells; bottom: in vivo application for radiography: (**a**) HER2-positive BT474 tumor; (**b**) HER2-negative MB-MDA231 tumor; (**c**) %ID/g of tumor estimated for 18F-labeled HER2 aptamer. “Adapted from [33] in accordance with the Creative Commons Attribution 4.0 International (CC BY 4.0) Creative Commons Attribution License”.

Gold nanoparticles, with the advantages of unique optical and electrochemical properties, surface plasmonic effect, considerable biosafety, and easy bioconjugation with Au-S chemistry, have been widely applied for designing targeted-delivery platforms [38]. AuNPs can be used as a carrier for the delivery of therapeutic or imaging agents to cancer cells or tissues. Mohammadinejad et al. introduced a nanocomplex for specific diagnosis and imaging of MCF-7 based on the detection of ATP molecules in cancer cells [13]. The nanocomplex was designed based on the conjugation of AuNPs with the MUC-1 aptamer (MUC1 apt-AuNPs) and conjugation of QDs with the ATP aptamer (ATP apt-QDs). As shown in Figure 1A, after adsorption of ATP apt-QDs on the MUC1 apt-AuNPs, the intensity of the QDs was quenched due to fluorescence resonance energy transfer (FRET) phenomena. In the following internalization of the complex in the MCF-7 cells through binding to the MUC 1 biomarkers on the MCF-7 cells, ATP apt-QDs can bind to ATP molecules, leading to separation from MUC1 apt-AuNPs and recovery of the intensity. 

Carbon-based nanoparticles, which mainly include graphene oxide (GO), carbon nanotubes (CNTs), and carbon dots (CDs), possess features of easy functionalization, low toxicity, great surface area, which is suitable for drug loading, and considerable in vivo stability. This feature makes possible the frequent use of carbon-based nanoparticles incorporated with aptamers as a biorecognition element. This platform can be a powerful platform for the aims of therapeutic and delivery systems. Benefiting from these materials, Mohammadi et al. designed a nanocarrier for siRNA based on the conjugation of an RNA aptamer of EpCAM with a single-walled carbon nanotube (SWNT)/piperazine–polyethylenimine non-viral vector. This platform was able to successfully suppress BCL9l in a positive EpCAM cell line MCF-7 [14]. 

QDs are assigned to the semiconductor nanoparticles containing periodic elements II/VI or III/V groups, with interesting features such as tunable size and emission (UV to near-infrared), high-intensity fluorescence intensity with narrow width, and considerable photostability [47]. Akbarzadeh et al. introduced a drug delivery and imaging platform based on loading the DOX on the silica-coated Gd-Zn-Cu-In-S/ZnS QDs/PEG/EpCAM DNA (Figure 1B) toward 4T1 and MCF-7 cell lines [15]. In vitro studies demonstrated the high toxicity of the platform for cell lines and the high efficiency of the proposed platform. Moreover, in vivo studies on the 4T1 tumor-bearing Balb/c mice proved the applicability of the designed platform in the prevention of tumor growth. 

Recently, silica nanoparticles have received huge consideration from scientists for designing targeted delivery due to the high porous structure for loading the therapeutic agents, easy modification, and high biocompatibility. Yang et al. synthesized a silica-based platform for the targeted delivery of DOX to CCRF-CEM human acute T lymphocyte leukaemia cells [16]. In this design, they used Sgc8 aptamer-conjugated mesoporous silica nanoparticles, and the results demonstrated a successful design, with good release and cell killing. 

The polymeric nanomaterials are assigned to a wide range of particles, such as micelles, hydrogels, and polymeric nanoparticles. The micelles are structured by self-assembling the copolymers with amphiphilicity features, which can be produced in a critical micelle concentration (CMC) [17]. Hydrogels with high hydrophilicity are designed from the cross-linking structure of polymers which possess high elasticity with considerable diffusivity of bifunctional molecules [48]. 

These nanocarriers, with the advantages of great drug loading and easy release, biocompatibility, and high biodegradability, are potent candidates for incorporation with aptamers for therapeutic and imaging aims. Polymeric nanoparticles include a wide range of hydrophilic polymers, such as polyethyleneimine (PEI), polyethylene glycol (PEG), polyethyleneimine (PEI), polycaprolactone (PCL), etc., which can be formed as nanoparticles [49] or a coating for other nanoparticles [14,19]. 

Benefiting from the conjugation of aptamers with polymeric nanoparticles, Zhao et al. introduced an irradiation therapy platform using AgNPs functionalized with PEG and aptamer As141 [19]. The proposed platform, with synergical advantages, showed an interesting radiosensitizing effect with a high rate of apoptotic cell death on the C6 glioma tumor cells. Moreover, in vivo studies showed more accumulation of synthesized nanoparticles in tumor tissues.

Liposomes are known as a phospholipid bag with a core made of aqueous media, which allows loading the drug into different parts of the core, bilayer, or interface layer. These nanocarriers have interesting features, including superior biocompatibility, hydrophilic drug delivery, and negligible toxicity [50]. Taking advantage of these nanocarriers, Li et al. introduced a liposome-based delivery system for loading the aptamer–DOX conjugation and delivering to the reversing drug resistance of MCF-7/Adr cells [18]. The nanoparticles successfully uptook with cells, and the entrance and release of the Ap-Dox complex was also successful. Afterwards, the interaction between aptamer AS1411 and nucleolin led to the entrance of Dox into the nucleus and prevention of drug efflux. 

Recently, the incorporation of aptamers with magnetit nanoparticles has been widely applied for targeted delivery systems due to super- and para-magnetism features, which enable drug delivery, magnetic resonance imaging (MRI), and hyperthermia therapy for cancer [51]. Sun et al. designed a targeted-delivery chemo-photodynamic platform using the conjugation of aptamer strands to the superparamagnetic iron oxide nanoparticles (SPION) [20]. The sequences of aptamers contained the AS1411 sequence with G-quadruplex folding and stem–loop folding. The nanocarrier sequences loaded 5, 10, 15, 20-tetra (phenyl-4-N-methyl-4-pyridyl) porphyrin (TMPyP) as a photosensitizer molecule and anticancer drug of daunomycin (DNM), respectively. The nanocarrier (TMPyP&DNM&Apt-S8@SPION) can show high cytotoxicity to nucleolin-overexpressed C26 and A549 cells by producing cytotoxic reactive oxygen species (ROS). 

Metal–organic frameworks (MOFs), with advantages of high porosity, crystalline-like structure, and synthetic adjustability, can be used in the different fields of biosensing [52], separation [53], energy saving [54], catalytic effects [55], thermal stability, and loading protection against enzymatic degradation [56]. Alijani et al. designed a core–shell Fe_3_O_4_@MOF nanocarrier (Figure 1C) using zirconium 2-amino-1,4-benzenedicarboxylate (UiO-66-NH2), MOF-loaded DOX, and CDs, and then conjugated it to AS1411 [21]. The proposed nanocarrier was successfully applied for selective delivery to triple-negative MDA-MB-231 human breast cancer cells, resulting in pH-dependent release, inhibition of cell proliferation, and induction of apoptosis. 

Due to some problems for in vivo imaging of photodynamic therapy and poor penetration of UV or visible wavelengths into deeper tissues for excitation of the photosensitizer (PS), in recent years, NIR-excitable up-conversion nanoparticles (UCNPs) have gained considerable attention in science due to excitation with NIR and emission of UV-vis wavelength. Also, they have some superior qualities, such as auto-fluorescent and background-free properties, deep penetration accessibility for excitation, and photochemical stability [57]. In this regard, Lin et al. introduced a NaYF4:Yb (20%), Er (2%) nanocluster (UCNP) for photodynamic therapy [22]. For this purpose, they used AS1411 to target the MCF-7 and Hella cells. In this design, a photosensitizer protoporphyrin IX was loaded on the aptamer, which can be excited by UV-Vis emitted from UCNP to produce the ROS. 

Recently, to apply aptamers for the evaluation of molecular and metabolic changes in living cells, a radiolabeled approach was proposed using radionuclide agent bound to the strand. Radionuclide agents can be gamma-emitting probes including indium-111 (111In), iodine-123 (123I), and 99mTc, and can be used in single-photon emission computed tomography tomography (SPECT) systems. Also, other agents include gallium-68 (68 Ga) or fluorine-18 (18 F), which can be used in positron emission tomography (PET). In this way, Kim et al. proposed 18F-fluoride- HER2 aptamers (Figure 1D) for in vivo molecular imaging [33].

### 2.2. Critical Note

The most important disadvantage of the nanoparticle-based targeted-delivery systems is the lack of nanoparticle clearance studies in the in vivo systems. So, it is proposed to add more details about biodistribution and clearance pathways in the future. Although most studies have used core–shell nanoparticles for targeted delivery, the administration of some nanoparticles such as QDs for in vivo studies may have some toxic effects on the bio-environment due to some inevitable heterogeneous distribution and leakage phenomena. So, it is better in the future for the long-term toxicity evaluation to be carried out for designed platforms which use nanoparticles, especially QDs in the formulation. Moreover, another problem is related to carbon-based nanoparticles, which benefit from a wide range of advantages but poor solubility and low dispersion in liquid media, which can limit the delivery systems. Also, another problem of some nanoparticles such as AuNPs is agglomeration in the culture media, which makes critical the condition of the targeted-delivery system. On the other hand, quenching and low intensity of fluorescent nanoparticles due to FRET or inner filter effect in the culture and biologic media can limit the activation of scientists [58]. Another limitation of the use of fluorescent nanoparticles like QDs for in vivo imaging is poor penetration of UV-Vis into deep tissues. Moreover, the wide range of UV-Vis may have a toxicity effect on the normal cells and tissues. So, the near-infrared (NIR) wavelength in the range of 700 nm to 1000 nm light, which possesses low phototoxicity, can be used [59,60,61]. For this purpose, UCNPs can be applied as a valuable luminescent alternative due to unique advantages with high contrast for in vivo imaging. However some disadvantages may limit the usage of UCNPs for clinical or preclinical procedures. One of the challenges is suitable excitation power to reach enough conversion luminescence and brightness, due to tissue damage. This may be resolved by modification of the cross-section of absorption and increasing the quantum yield. Moreover, the use of an excitation wavelength of more than 980 nm can resolve the probability of tissue damage [62]. Another challenge can be providing a microscope equipped with an external 980 nm laser, which can increase the price and accessibility.

Another alternative for imaging can be a radio-labelled aptamer, which can use lower energy for the excitation of radioactive material. Although this technology can be used for killing and slowing the cancer cells’ growth and imaging, the use of this material has some inevitable problems for human health. Some of these side effects are feeling exhausted, memory problems and distraction, hair loss, skin defects, and vomiting [63]. 

The other limitation of the application of fluorescent or radioactive-labelled aptamers in tracing the targeted delivery to cells is the non-identification of the resulting signal from the entire cell population or a cell subset. So, the qualitative imaging method by confocal microscopy, a popular method, has been applied for the evaluation of internalization to cells, which makes it impossible to count a low number of cells [64]. Moreover, the fluorescence bleaching of labelled aptamers by high-intensity laser can make cell counting more difficult. To resolve this problem, the application of high-quantum-yield fluorophores, such as QDs and Alexa Fluor dyes, for labelling the aptamer, and more expensive methods, such as LED-based systems or flow cytometry systems, have been progressed [65]. 

Although the proposed platforms for targeted delivery show high sensitivity toward the considered cell, it may be better if some other studies are performed in the future regarding scalability for entrance to cells and comparison with other clinical methods for assessment of applicability and evaluation of the entrance of the nanocomplex to the tissues and the release of therapeutic or imaging agent. The most important problem of the studies [15] with pH-sensitive release claims is low release (e.g., 15%) after 24 h under acidic conditions, which can be critical for drug delivery systems. Also, in vivo stability is another problem of past studies [16], so more studies must be conducted in the future. Although the covalent binding of the aptamer to nanoparticles possesses the advantages of strength and stability in different pH and temperature conditions, the problem of probable denaturation during chemical reaction for covalently binding may limit its applicability. These phenomena lead to the poor functionality of aptamers and less efficiency of the targeted delivery system [66]. In this way, incorporation of aptamers with liposomes can be performed both noncovalently and covalently. The former can be created by electrostatic interaction with cationic liposomes and the latter can be accomplished through chemical conjugation, but both methods lead to placing the aptamer in the core or on the surface of liposomes. Moreover, the stability of the encapsulation with liposomes in various physiological conditions must be evaluated, which the study of Li et al. [18] lacks. 

Although the application of magnetite nanoparticles for targeted delivery possesses extraordinary features, there are some disadvantages, such as the difficulty of using outer magnetic field for delivery of magnetite nanocarriers to organs, limitations of frequency, exposure time, and intensity of magnetic field for patients [67]. 

Application of MOF possesses some deficiencies which may limit its applications, including weak stability, which may lead to prompt release and leakage of drugs or high stability in aqueous media, which can reduce the percentage of drug release and efficiency of the method. Moreover, due to the lack of metal–carboxylate sites, which can be necessary for modifications, some pretreatments must be performed for MOF, which may lead to structural failures and material amorphization [68]. 

### 2.3. Aptamer–Drug Conjugation (ApDC) 

Aptamers, with advantages of high stability, reversible conformation, powerful targeting, easy conjugation and modification, and programable conformation, can be used as a carriers for the ApDC technique. This technique possesses three main members, includes the aptamer as a ligand for targeting and delivery to sites or biomarkers, a warhead, which is a therapeutic agent (drug), and a linker, with the function of loading the therapeutic agent [69,70]. Depending on whether the linker is bonded covalently or noncovalently, two modes of physical conjugation or intercalation and a chemical linker can be described. 

#### 2.3.1. Physical Conjugation (Intercalation)

The DNA nanostructures are known to be biodegradable carriers with high biocompatibility, and they do not need any elements for internalization into the cells; they possess more bio-friendly features in comparison with inorganic and polymeric nanomaterials. Due to the folding ability of aptamer the anticancer drugs, mainly the anthraquinone family, and especially doxorubicin (DOX), can intercalate in between bases of the DNA double helix. The intercalation can be through opening the deoxyribose–phosphate and laminated interaction with the plane aromatic base. The intercalation process leads to unzipping two strands of dsDNA, and after internalizing, the intercalated agent can be released. This process can be reversible if the DNA structure is not destroyed. This method is easy to perform, without any chemical synthesis process, and inexpensive [71,72].

In this way, icosahedral DNA complexes were formed and used as nanoparticles for carrying doxorubicin toward MCF7 cells [23]. The 3D DNA nanostructures were formed by assembling five DNA strands to form a five-point-star motif. The internalization of cells happens through the recognition of MUC1 on the surface of cells. DOX was able to release from DNA nanostructures in the acidic medium of lysosomes, resulting in the prevention of side effects to a considerable extent and increasing effectiveness. 

Taking advantage of DNA complexes, Liu et al. designed a similar nanostructure that includes a DNA aptamer with G-quadruplex structure for interaction with the MUC1 biomarker on the MCF 7 cell surface and double-stranded DNA for loading DOX [24]. 

In another study by Lopes et al., a delivery system was designed based on the conjugation of the pancreatic cancer RNA aptamer P19 to therapeutic agents such as gemcitabine, 5-fluorouracil (5-FU), monomethyl auristatin E (MMAE), and a derivative of maytansine 1 (DM1) [25]. The results demonstrated that delivery systems were able to inhibit the proliferation of PANC-1 and AsPC-1 cell lines with the mechanism of phosphorylation of histone H2AX protein on Ser139 (γ-H2AX). Moreover, mitotic G2/M phase arrest was the mechanism of action of delivery systems of MMAE and DM1.

**Figure 2:**
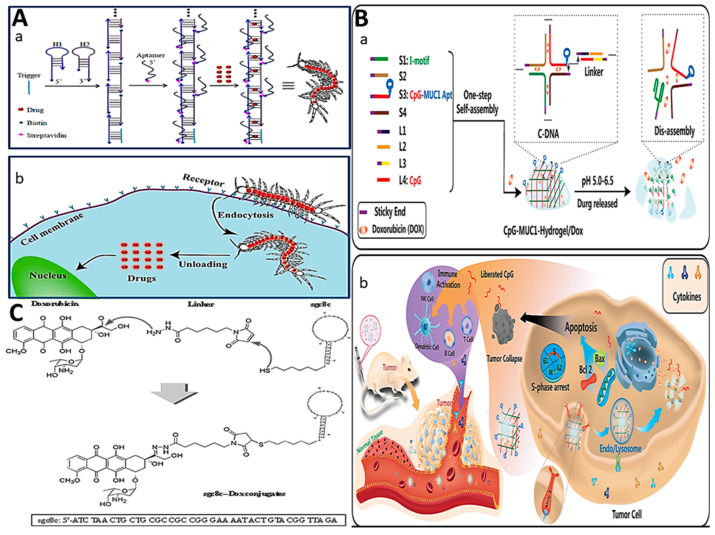
(**A**) Application of HCR in drug delivery. (**a**) Formation of nanocentipede and loading of DOX; (**b**) eternalization of nanocentipede–DOX system into SMMC-7721 cell. “Reprinted from [27] with permission from ACS”. (**B**) Application of cross-hybridization formation of DNA (C-DNA). (**a**) Formation of CpG-MUC1-hydrogel for (**b**) targeted drug delivery to cells with MUC1 biomarkers. “Reprinted from [28] with permission from ACS”. (**C**) Covalent binding of sgc8ca to doxorubicin (DOX) through hydrazone (linker). “Reprinted from [26] with permission from John Wiley and Sons”.

Li et al. introduced an innovative design based on hybridization chain reaction (HCR) with scaffold formation for delivery of DOX to SMMC-7721 cells [27]. In this design, a short DNA triggered the self-assembly process of biotinylated hairpin DNAs (Figure 2A). After the formation of scaffold hybridization, streptavidin-aptamers (Zy1) were able to conjugate to hybridize through streptavidin–biotin binding. The DOX was loaded on the scaffold hybridization body and aptamers Zy1 as legs were used for targeting. 

Benefiting from the one-step self-assembly method, a cross-hybridization formation of DNA (C-DNA) nanohydrogels was designed using eight ssDNA [28]. As shown in Figure 2B, this nanostructure is formed by S1–S4 DNA sequences and linker L1–L4 DNA sequences, which mainly include unmethylated cytosine-phosphate-guanine oligonucleotides (CpG ODNs), I-motif cytosine (C)-rich single-stranded DNA with quadruplex formation ability in acidic conditions, and MUC1 aptamer. After internalization in cells, under acidic conditions, the I-motif tends to form a quadruplex, leading to the dissembling C-DNA and release of DOX. 

#### 2.3.2. Covalent Conjugation 

Covalent and chemical bonding of drugs to the aptamers has been widely used for designing drug delivery systems due to the advantages of simple modifications of aptamers and easy cleavage of drugs. The linkers can be non-cleavable or cleavable. The cleavage of linkers can be dependent on the enzymatic reaction, pH, and temperature. But non-cleavable linkers are usually applied when the therapeutic agent is a sequence, such as small interfering RNAs (siRNAs) or nucleotide analogues and nucleobases, which are conjugated to target aptamers through a sequence [32]. 

There are three kinds of chemical linkers which are usually applied for the conjugation of drugs: hydrazone and thiol-to-thiol and dipeptide bonds. Hydrazone is formed by the reaction of hydrazine on aldehydes and ketones with a hydroxyl group. This linker can be dissociated by hydrolysis in acidic conditions of endosomes and lysosomes [73]. The thiol-to-thiol linker can be used for bonding thiolated drugs with thiolated aptamers. Sometimes to increase stability, the thiolated maleimide or PEG can be used between the drug and aptamer. The disulfides can be decomposed in acidic conditions of endosomes. The dipeptide bonds which are created between amino acids, such as valine-citruline (Val-Cit) and phenylalanine-lysine (Phe-Lys), can specifically be decomposed by Cathepsin B as a lysosome protease, which is overexpressed in cancer cells. 

Nucleotide analogues and nucleobases with different antiviral, immunosuppressive, and anticancer functionalities [74,75] can be inserted as a sequence in aptamer strands. In this design, both functionalities of targeting the aptamer and the therapeutic effect of the nucleotide analogue strand can be retained to a high extent with more safety in comparison with chemical linkers.

Huang et al. designed a targeted drug delivery system using sgc8ca apt-DOX conjugated to tumor cells [26]. In this study, the DOX was conjugated to the aptamer through hydrazone binding while the applicability and naturality of the aptamer were maintained (Figure 2C). The results of several assays such as selective internalization and toxicity confirmed successful targeted drug delivery to tumor cells and specifically killing of the human T-cell ALL (CCRF-CEM) and human B-cell Burkitt’s lymphoma (Ramos) cell lines. The evaluation of fluorescence intensity obviously proved the successful uptake of conjugation to cells. After recognition of proteins in the cells, the drug can be released from the acid-labile linkages inside the endosomes to transport to nuclei. 

Benefiting from linker-based therapeutic agent binding, Wang et al. introduced a therapeutic conformation to low-density lipoprotein receptor (LDL-R) as a biomarker target on the surface of cancer cells, such as Huh-7 liver cancer cells and MDA-MB-231 breast cancer cells [29]. In this design, antimiR-21 DNAzyme was linked to the aptamer through a bridge containing five sequential deoxythymidine (d-TTTTT-) to retain the applicability of both aptamer and DNAzyme. The results showed that the expression of miR-21 decreased up to 56%.

In this way, paclitaxel (PTX) with a hydroxyl group at the 2′ position of PTX was covalently bound to the nucleolin aptamer (NucA) through a dipeptide (S-S) bond [30]. After the entrance of NucA- PTX to tumor cells, the linker was cleaved through cathepsin as intracellular lysosomal protease leading to the release of PTX. 

Also, Theil et al. introduced a delivery system based on the conjugation of HER2 aptamer to siRNAs targeting the anti-apoptotic gene, Bcl-2. This system was able to potentially internalize the HER2-positive cells (N202.1A cells) to silence and prevent Bcl-2 gene expression [32]. 

Benefiting from covalently binding, HER2-specific aptamer was conjugated to the mertansine (DM1) through disulfide bonding between DM1 and the 3′ end of the aptamer, which can be cleaved in early and late endosomes [31]. This cleavage can increase the anticancer activity of the drug through the facilitation of DM1 release from the endocytic pathways. Moreover, in this design, the PEG molecule was bound to the 5′ end of aptamer to increase of biocompatibility and circulation ability in vivo. In this study, the aptamer–DM1 conjugation was used for in vivo studies of mouse xenografts with BT-474 breast cancer.

#### 2.3.3. Critical Note 

The studies demonstrated that the incorporation of the aptamer and drugs has been formed as well for targeted delivery to the cells. This promising technique is potentially more applicable in the human treatment domain provided that a lot of completed procedures and assays are carried out. Although these studies have been very valuable, they may be limited due to some disadvantages. In most studies, the characterization and synthesis of the aptamer–drug conjugation is not well-detailed. Although studies reported successful conjugation for targeted drug delivery, some disadvantages, such as the in vivo applicability and cancer treatment effects, including immune system reactions, should be tested. Most papers deal with in vitro evaluation to validate the applicability of delivery systems. Also, it is better to further discuss the off-target, toxicity, and side effects of the designed ApDC on the normal cells or tissues [12]. Moreover, covalent bonding requires the chemical process and reagents, which may reduce the nature of the aptamer and increase the toxicity of the delivery system. Furthermore, other challenges could include the low amount of drug loading, expensive reagent requirements, and several boring steps required for covalent conjugation. On the other hand, physical conjugation of drugs may face the loading challenge, especially in the in vivo environment, which contains a lot of macromolecules and interferences, leading to the release of drugs, low loading, and low efficiency of drug delivery. Moreover, probable changes in the formation and folding during processes, which contain different media and conditions, can be inevitable, leading to low selectivity and failed efficiency. To evaluate the therapeutic efficacy of results, the designed delivery system can be replicated in other animal models or even human clinical assays. 

## 3. Biosensors

The timely diagnosis of cancers, which is an inseparable part of fast therapeutic aims, has a central role in the improvement of community health. To diagnose cancers and biomarkers, a lot of traditional methods such as enzyme-linked immunosorbent assay (ELISA), polymerase chain reaction (RT-PCR), flow cytometry, and mass spectrometry [76] have been applied. Unfortunately, the aforementioned methods suffer from some disadvantages, such as high cost, requirements of many experts, and being time-consuming. So, the development of simple, sensitive, easy-to-operate techniques is urgent. Nowadays, biosensors can be a potent alternative to traditional methods. Biosensors are known as a potent diagnosis technique for the detection of a wide range of molecules including proteins, toxins, cells, bacteria, DNAs, miRNAs, etc., which are important in different fields of medicine, food, and the environment. Biosensors can record and translate the biological response to readable signals through the transduction system during processes of collection and amplification of the signal. In terms of the manner of translation, different biosensing systems can be introduced, such as optical, electrochemical, piezoelectric, thermometrical, and magnetical [66]. Biosensors should be designed so that they can diagnose the biomolecules with high selectivity among the interferences of biological systems. Recently, several biosensors have been designed for diagnosis of cancers through detection of biomarkers in medical sciences. In this way, various sensors have been fabricated using bioreceptors such as antibodies, enzymes, aptamers, etc. Among them, aptamers have received high attention due to some advantages such as small size (12–30 KDa), wide range of targets, simple synthesis, high stability, easy modification, long-term storage, low cost, and low immunogenicity [77]. In recent years, the point-of-care (POC) technique has been pervasive for clinical aims. The usual medical assays which are centralized in clinical or hospital labs need a large amount of samples and a long time for the interpretation of results. POC devices enable person-centered assays in a short time and require fewer samples, enabling cheaper tests in emergency conditions [78]. Due to the unique features of aptasensors, they can be used for the development of POC devices. Concerning the kind of the platforms, aptasensors, mainly designed for cancer cell lines, can be classified into optical, electrochemical, paper-based, microfluidic, and smartphone-based. The summarized data of studies are shown in Table 2.

Optical platforms contain three main members, including the light source, the sensor of the target (light), and a detector, which collects the light from the sensor and evaluates it. Optical biosensors, with advantages of high sensitivity, simplicity, and easy miniaturization, have been highly desired for incorporation with aptamers. 

**Figure 3:**
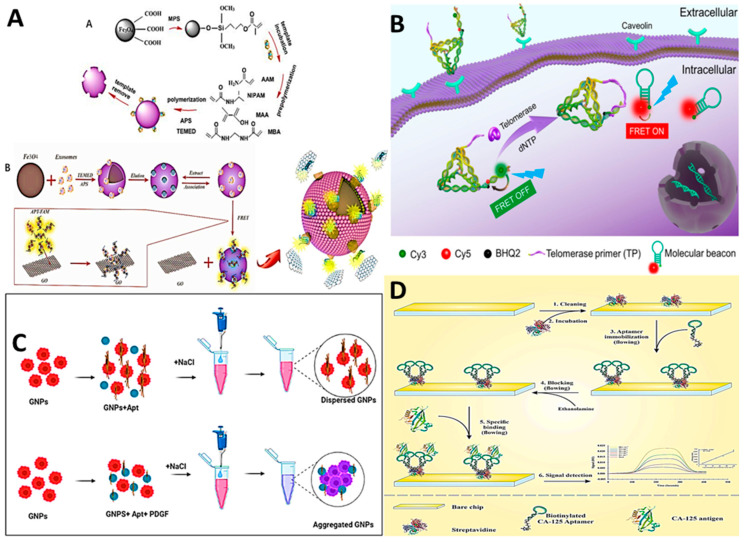
(**A**) Optical aptasensors. (**B**) Optical aptasensors FRET-based; (**C**) colorimetric; (**D**) SPR. “Reprinted from [79,80,81,82] with permission from Elsevier”.

In this way, fluorescence resonance energy transfer (FRET)-based aptasenors have been frequently designed for the detection of cancer cell biomarkers. FRET, as a nonradiative phenomenon, usually happens through the transmission of energy of a fluorophore as a donor to an acceptor, which may able to emit the energy to a longer wavelength [88]. Benefiting from this phenomenon, several biosensors have designed FRET biosensors based on energy transfer between the fluorophore-conjugated aptamers and GO as a quencher. As an example, in this field, Feng et al. designed a FRET system based on the selective adsorption of exosomes on molecularly imprinted polymer (MIP)-coated Fe_3_O_4_ nanoparticles followed by competition with aptamers in the GO/aptamer-FAM system (Figure 3A) [79]. This competition led to the release of aptamer-FAM and the recovery of fluorescence intensity. 

Taking advantage of aptamer-incorporated FRET, molecular aptamer beacon (MAB), which possesses both fluorophore and quencher moieties at two ends of the strand, has been introduced with the aim of imaging and quantification. The design of the molecule is based on switching the folding of the stem–loop structure to recognize the target by on/off yielding [89]. Yue et al. designed an interesting targeted-delivery system combining tetrahedral DNA nanoprobe (TDNp) and MAB, which contains fluorophores Cy3 as donor and Cy5 as acceptor [80]. As shown in Figure 3B, in this system, TDNp is prepared from L1–L4, which includes a primer (TP) at the strand L2 end and a hybridization section for MAB at L3. The delivery was able to internalize to the cell through the pathway of caveolin-mediated endocytosis. In the absence of telomerase, after hybridization and folding change, the Cy3 and Cy5 can stay at a far distance, leading to FRET inhibition. But the presence of telomerase led to the duplication of TP at the 3’ end of L2, which extended to L3 with consecutive TTAGGG sequences. The hybridization with L3 resulted in the release of MAB and FRET phenomena. 

Nowadays, colorimetric biosensors, with the advantages of being easy to use, simple interpretation, low cost, visibility, etc., have been frequently incorporated with biomarker detection. In this way, AuNP-based colorimetry has been widely applied for designing this kind of sensor through the color change from red to purple, which happens through the aggregation of AuNPs and variation in wavelength at 520 nm. Dispersed AuNPs possess an inter-nanoparticle distance more than the particle diameter, which can seem red, but in aggregation form, this distance becomes lower, leading to purple [90]. The most interesting design of a AuNP–aptamer colorimetric biosensor employs the aggregation that results from adding salt. In this design, the aptamer remained among AuNPs, preventing aggregation in the presence of NaCl salt. By adding the target, it can be bind to the aptamer, leading to conformation changes and poor defense of AuNPs against aggregation. Hasan et al. designed this kind of biosensor for the detection of platelet-derived growth factor (PDGF) (Figure 3C) [81]. 

Surface plasmon resonance (SPR) can occur through nanoplasmonic phenomena using noble metal nanoparticles, which can focus the light into nanoscale regions. This incidence of light leads to the increase in collective oscillation, which is produced by surface free electrons. This phenomenon results in the scattering or absorption of light, leading to the plasmon resonance frequency change. This technique includes the advantages of label-free possibility, low cost, and easy sample preparation [91]. Taking advantage of SPR, Shahbazlou et al. introduced an SPR platform (Figure 3D) designed through the immunization of CA125 aptamer, through streptavidin–biotin, on a bare gold chip [82]. This design was used for the detection of CA125 in human serum samples. 

**Figure 4:**
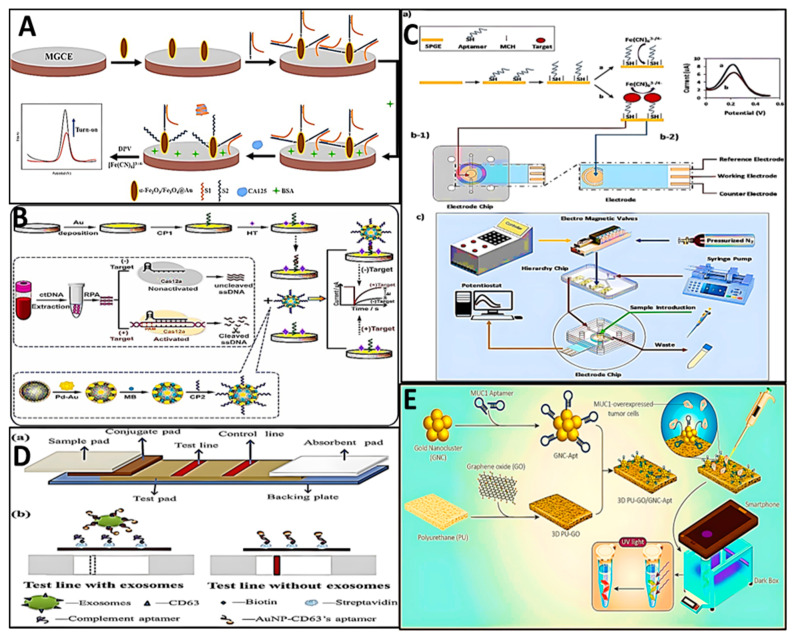
Designing of aptasensor for cancers. (**A**) Label-free electrochemical sensor; (**B**) Cas12a/crRNA-based electrochemical sensor; (**C**) microfluidic: (**a**) designing and detection of target on electrode; (**b-1**) inserting the electrode in chip; (**b-2**) immobilization of aptamer on the electrode; (**c**) designing of microfluidic system; (**D**) LFA; (**E**) smartphone. “Reprinted from [83,84,85,86,87] with permission from Elsevier”.

Electrochemical biosensors which synergically contain the sensitivity of the electrochemical transducer and specificity of bioreceptors can be classified into biocatalytic and affinity sensors according to the kind of biorecognition element. The biocatalytic sensors usually use enzymes, tissue slices, etc., and affinity sensors use antibodies, aptamers, and DNA [66]. Among different kinds of electrochemical sensors, aptasensors have frequently been applied due to their simple design, sensitivity, and low price. According to labelling with an electrocatalytic agent, aptasensors are classified as label-free and label-based. Ni et al. designed a label-free aptasensor for cancer antigen 125 (CA125) by modification of the electrode using magnetic α-Fe_2_O_3_/Fe_3_O_4_/Au nanocomposite followed by immobilization of the partial complementary strand on the electrode. As shown in Figure 4A, in the presence of CA125, the aptamer is released from the complementary strand, and the current is increased [83]. Moreover, recently, a new generation of electrochemical sensors was developed based on the CRISPR-Cas systems. Cas12a nuclease is known as a powerful tool for the detection of nucleic acid strands and is known as an RNA-guided DNase which possesses the advantages of considerable sensitivity and specificity, and efficient cutting ability. Cas12a, which is directed by CRISPR RNA (crRNA), contains T-rich protospacer-adjacent motif (PAM), creating a Cas12a/crRNA complex to specifically detect DNA with *trans*-cleavage and *cis*-cleavage activities to discriminate ssDNA or detect DNA. Taking advantage of this technique, Liu et al. designed an electrochemical biosensor for the detection of EGFR L858R as a circulating tumor DNA (ctDNA) in lung cancer [84]. In this design, MB/Fe_3_O_4_@COF/PdAu bound to a complementary strand and another complementary strand was immobilized on the electrode. As shown in Figure 4B, the resulting ssDNA from Cas12a *trans*-cleavage activity can be hybridized by complementary strands, leading to the increase in the MB signal. 

To improve the sensitivity of biosensors, the incorporation with microfluidic systems has always been a good idea for scientists due to the enhancement of mass transfer to the surface of biosensors. The microfluidic technique can donate valuable features to the biosensors, such as low sample requirements, high throughput and fast detection, miniaturization, and portability [92]. Taking advantage of a microfluidic assay, Khaksari et al. introduced a platform for electrochemical detection of A549 cells. In this design, a screen-printed gold electrode was modified by integrin α6β4-specific DNA aptamer (IDA) and inserted in on-chip gas-actuated microvalves for the microfluidic platform to provide the flows (Figure 4C) [85]. 

Lateral flow assay (LFA), as a paper-based technique which possesses valuable advantages including low cost, simple design, and visual results interpretation, can be used as a POC device for the diagnosis of cancer [93]. It can be used for the detection of a wide range of targets such as bacteria, toxins, hormones, biomarkers, etc. Recently, the application of aptamers instead of antibodies has been pervasive due to the superior advantages of aptamers over antibodies, which was discussed in the preceding sections. The LFA strips contain the sample pad for sample loading, the conjugated pad for loading the labelled bioreceptors, the test pad, which is a nitrocellulose (NC) membrane, for capillary migration of sample and separation, the absorbent pad for absorption of buffer and sample, and the support back for retaining the components of LFA strips. Moreover, the NC is normally coated with two lines, including the test line (T-line) and control line (C-line). The T-line usually can dispense the target bioreceptor, and the C-line is also a bioreceptor for the labelled bioreceptor [94]. As shown in Figure 4D, Yu et al. fabricated a lateral flow test strip for the diagnosis of identification of CD63 on non-small cell lung cancer (NSCLC) exosomes [86]. In this design, they dispensed streptavidin (SA)-biotin-CD63 aptamer, complementary on the T-line, and the AuNP-CD63 aptamer was used as a probe. 

The smartphone is known as a new generation of POC devices which possess high integrability with different types of biosensors, simple design, and portability. This kind of biosensor enables control of the reaction process of biosensors with simple software on the smartphone [95]. Sanati et al. designed a lab-in-a-tube technique for the detection of MUC1 on the circulating tumor cells (CTCs) using gold nanocluster (GNC)-aptamers as emitter and polyurethane (PU) coated with GO as quencher [87]. As shown in Figure 4E, they designed the dark chamber to be equipped with UV-LED emitters, an aluminum heat sink for the release of heat, and a cylindrical chamber for the reaction tube, which can be controlled through a smartphone with ImageJ software from the top of the chamber.

## 4. Critical Note

Although designed biosensors can be introduced as the most potent technique for the early diagnosis of cancer, some disadvantages and failures, such as the inaccessibility of some methods for public and non-expert persons, may prevent the development of these methods as POC devices. For example, the microfluidic device, which was designed by Khaksari et al. [85], resulted in acceptable sensitivity, but the design of this platform required large and expensive equipment, which can only be used by specialized experts. Also, FRET-based techniques, which are based on the transmission of energy, may suffer from autofluorescence interferences or FRET between fluorophore and macromolecules in biological samples, leading to negative or positive errors. Colorimetric methods based on AuNPs are seriously exposed to aggravation in biological samples. On the other hand, the sensitivity of some techniques, like MAB, are highly dependent on the aptamer folding and may be changed by conformation and changes in 3D folding in different conditions, depending on the pH, solutions, and temperature. Moreover, LFA and smartphones, although providing some favorable conditions such as simplicity and accessibility for the development of POC techniques, show low sensitivity and a lack of validity of results, which may affect the development of potent POC techniques. Some enzymatic methods, such as Cas12a, may suffer from instability in the long term, which leads to poor accuracy of biosensors. The electrochemical and SPR sensors are the most important, reliable, and accurate techniques and have the potential of miniaturization for accessibility and the label-free ability for the use of bioreceptors. The hand-held platforms, with easy interpretation of results and low price, can be designed using these techniques, which possess a heightened role in healthcare and treatment. 

## 5. Conclusions and Future Perspective

This review potentially demonstrates that aptamers have been successfully introduced as a therapeutic and diagnostic tool for designing imaging, drug delivery, and biosensors due to their unique features, including high selectivity, affinity, and compatibility with different methods. The targeted-delivery technology significantly benefited from aptamer advantages for efficacy in cancer therapy and decrease in toxicity. Studies demonstrated that aptamers potentially played the role in targeting and as nanocarriers for drug delivery systems with drug intercalation, chemical linkers, and also nucleotide analogues. There are still some challenges in aptamer-based targeted-delivery systems, such as fast clearance from the body and kidney excretion, nucleus degradation, and in vivo thermal instability [96]. Moreover, drug–aptamer conjugation is mostly limited to DOX use, which can easily intercalate into the nucleic acid strands. So, developing the delivery systems with different drugs could be the most important challenge to be evaluated in the future. This review demonstrated that the incorporation of aptamers with biosensor platforms was implemented as well to produce accurate, selective, and sensitive diagnosis tools for biomarkers. But the most important shortcoming of these biosensing platforms is their inaccessibility, and most of them remain limited to articles and studies. So, it is very important to develop POC devices for cancers which are accessible to the public, like glucometers or pregnancy checkers. Although it is mentioned that aptamers possess superior features in comparison with antibodies for therapeutic aims, the application of aptamers is limited, and this must be addressed in the future. 

This review demonstrated that the aptamer is well-incorporated with in vitro and in vivo assays for diagnosis and therapy of different kinds of cancers in preclinical stages. Although a lot of commercialized aptamer-based drugs have been introduced in recent years, there has also been a considerable desire for biomarker diagnosis and therapeutic aims using antibodies in clinical assays. The main reason for distrust of the implementation of aptamers in clinical assays may be some defects in SELEX stages due to some inaccuracy in the process, impurity of targets, and mostly in vitro synthesis, which lead to the lower selectivity and interaction with other in vivo targets. One of the powerful and effective techniques which can be applied for resolving the poor tissue permeability problem is in vivo SELEX, which can be directly performed in live media [97]. In this technique, nucleic acid strands, which are nuclease-resistant, are injected intravenously into tumor-bearing mice followed by separation of tissue, isolation of nucleic acid strands, and in vitro amplification. This strategy is assumed to create an effective targeted-delivery system which enables the diagnostic and therapeutic aims in clinics. Unfortunately, this technique may suffer from some disadvantages, which may limit clinical applications, such as differences in specificity, inability to amplify polymerases, costly extraction, and unreliability of in vivo screening. So, future developments in this area must be focused on the low-cost, fast, and improved identification after screening. 

Another suggestion for future SELEX processes is making automation possible, which can reduce the errors and increase reproducibility. This concept can make the presence of experts unnecessary and reduce the time required for assays and reagents, while the throughput can be decreased. The automation can be performed in two forms, that is, unit automation and full automation. The differences between these methods involve human interference in some (or no) stages of the pipeline. The unit automation design possesses the advantage of possible modification of molecules during some stages of the pipeline [65].

In the next few years, some technologies, such as single-molecule or cell screening, identification, and sequencing, could be very promising for resolving the problems. Moreover, artificial nucleotide bases can be developed to increase the biostability of screened aptamers for clinical assays. The detailed information demonstrated that nanoparticles possess an undeniable role in diagnosis and therapeutic aims. But some conditions, such as size, low distribution, and unpredictable behavior of materials in in vivo media make critical their production and usage in clinical assays. Nanomaterials are mainly assigned to unbound particles with a size distribution of <100 nm, where at least 50% of particles must meet that condition without agglomeration. So, it is required to perform safe-by-design assays for clinical development due to changes in physicochemical and magnetic properties in the range of <10 nm and also biological properties in the range of > 200 nm [98]. Moreover, another source of anxiety in this field is the toxicity of nanoparticles in the body, which may remain unresolved in the future. In particular, the aptamer–nanoparticle conjugations require chemical agents, which increase the possibility of toxicity for the body. The reported information by articles and studies contains some characterizations which cannot be used as a citable reference for companies to produce diagnostic and therapeutic products. Some efforts in this field have been performed for the evaluation of material toxicity, which is more limited to cosmetics without any nano-toxicology evaluations. So, in future, more efforts are expected to be carried out in in vivo studies, including computational biology, biostability, side effects, nanotoxicity, coating, biodegradability, biocompatibility, dose of therapeutic agent incorporated with size, evaluation of penetration and diffusion, etc.

Biotechnology companies must focus on the evaluation of and reduction in the toxicological effects of nanoparticles to produce medicinal products. Moreover, drug delivery studies should describe and explain predictive effects more than simple reports of results. Recently aptamer-based CRISPR-Cas, as a new generation of biosensors, has gained the attention of scientists for the diagnosis of biomarkers and toxins. This new design of sensors is a promising field which can be helpfully and powerfully applied in different fields such as bioanalytical sensors, molecular biology, and enzymatic engineering. Although some shortcomings, such as complicated processes, optimization of acting conditions of Cas proteins, and highly expert requirements, may limit its application and accessibility, due to high accuracy, this method must be further developed and incorporated with electrochemical methods for designing handheld and smartphone platforms which can be easily used in clinics.

## Figures and Tables

**Table 1:** The summarized information aptamer-based targeted-delivery systems.

Method of Delivery	Sequences of Aptamer (5′ to 3′)	Cell or Animal	Marker	Therapeutic Agent	Ref.
Nanocomplex of ATP aptamer/QDs and MUC-1 aptamer/AuNPs	ATP-aptamer: NH2-AACCTGGGGGAGTATTGCGGAGGAAGGTMUC1-aptamer: 5′-SH-GAAGTGAAAATGACAGAACACAACA-3′	MCF-7	Muc-1, ATP	-	[13]
EpCAM aptamer-conjugated SWNT/piperazine–polyethylenimine	EpCAM aptamer: GCG ACU GGU UAC CCG GUC G SiRNA: GGAUGUUCAAGAUCCCAUGCAGCTC	MCF-7	EpCAM	siRNA for suppressing BCL9l	[14]
silica coated-Gd-Zn-Cu-In-S/ZnS QDs/PEG/EpCAM DNA	EpCAM aptamer: CAC TAC AGA GGT TGC GTC TGT CCC ACG TTG TCA TGG GGG GTT GGC CTG	4T1, MCF-7	EpCAM	DOX	[15]
Sgc8 aptamer-modified silica nanoparticles system	Sgc8 aptamer: ATCTAACTGCCGCCGCGGGAAAATGTACGGTTA G(T)10-COOH	CCRF-CEM human acute T lymphocyte leukemia	protein tyrosine kinase-7 (PTK-7)	DOX	[16]
As141 aptamer-conjugated pluronic F127/beta-cyclodextrin-linked poly (ethylene glycol)-b-polylactide block copolymers (β-CD-PELA)	As141 aptamer: TTGGTGGTGGTGGTTGTGGTGGTGGTGG	MCF-7, female BALB/c nude mice	Nucleolin	DOX	[17]
Encapsulation of aptamer-DOX in liposome	Aptamer AS1411: GGT GGT GGT GGT TGT GGT GGT GGT GGT T	Human breast tumor MCF-7/Adr cells	nucleolin	DOX	[18]
irradiation therapy using AgNPs functionalized with PEG and As141 aptamer	As1411 aptamer: (CH2)6-NH2-GGTGGTGGTGGTTGTGGTGGTG GTGG	C6 glioma, C6 glioma-bearing mice	nucleolin	-	[19]
Photodynamic therapy by aptamer-conjugated superparamagnetic iron oxide nanoparticles (SPION) loaded by daunomycin (DNM) and 5, 10, 15, 20-tetra (phenyl-4-N-methyl-4-pyridyl) (TMPyP)	As1411 and DNM aptamer: 5′-NH2-GGG GGG GGT TGT CCC CCC CCT TTT TTG GTG GTG GTG GTT GTG GTG GTG GTG G	C26, A549	nucleolin	DNM, TMPyP	[20]
Fe_3_O_4_@ UiO-66-NH2 MOF/DOX/CDs/AS1411 aptamer	As1411 aptamer: GGTGGTGGTGGTTGTGGTGGTG GTGG	MDA-MB-231	nucleolin	DOX	[21]
photosensitizer protoporphyrin IX/ AS1411/NaYF4:Yb, Er nanocluster (UCNP)	As1411 aptamer: NH2–GG TGGTGGTGG TTG TGG TGGTGG TGG	MCF7, Hella	nucleolin	photosensitizer protoporphyrin IX produced ROS	[22]
assembling five DNA strands to form five-point-star motif	strand I: ATAGTGAGTCGTATTAATTAACCCTCACTAAAAAGGATCCGGATCCTT strand II: TTTAGTGAGGGTTAATCATACGATTTAGGTGAAAGGATCCGGATCCTT strand III: TCACCTAAATCGTATGGGAGCTCTGCTTATATAAGGATCCGGATCCTT strand IV: ATATAAGCAGAGCTCCTAGAAGGCACAGTCGAAAGGATCCGGATCCTT strand V: TCGACTGTGCCTTCTATAATACGACTCACTATAAGGATCCGGATCCTT	MCF7	MUC1	DOX	[23]
Assembling two strands containing a DNA aptamer with G-quadruplex and double-stranded DNA	CCCCCCCCCCTGTTGGGGGGGGGGTTTTTTTTTGGTGGTGGTGGTTGTGGTGGTGGTGG	MCF7	MUC1	DOX	[24]
Conjugation of aptamer P19 to gemcitabine, 5-fluorouracil (5-FU), monomethyl auristatin E (MMAE) and derivative of maytansine 1 (DM1)	GGGAGACAAGAAUAAACGCUCAAUGGCGAAUGCCCGCCUAAUAGGGCGUUAUGACUUGUUGAGUUCGACAGGAGGCUCACAACAGGC	PANC-1 AsPC-1	cells	Gemcitabine 5-FU MMAE DM1	[25]
Covalently binding sgc8ca aptamer–doxorubicin	ATC TAA CTG CTG CGC CGC CGG GAA AAT ACT GTA CGG TTA GA	Human T-cell ALL (CCRF-CEM), human B-cell Burkitt’s lymphoma (Ramos)	kinase 7 (PTK7)	DOX	[26]
self-assembly of biotinylated hairpin DNAs followed by streptavidin–aptamer (Zy1) conjugation	H1: Biotin-CGT CGT GCA GCA GCA GCA GCA GCA ACG GCT TGC TGC TGC TGC TGC TGC H2: Biotin-TGC TGC TGC TGC TGC TGC ACG ACG GCA GCA GCA GCA GCA GCA AGC CGT Trigger: TGC TGC TGC TGC TGC TGC ACG ACG Zy1: ACG CGC GCG CGC ATA GCG CGC TGA GCT GAA GAT CGT ACC GTG AGC GCG T(T)_10_- streptavidin	SMMC-7721	cell	DOX	[27]
self-assembly of 8 sequences of S1–S4 DNA and linker L1–L4 DNA to form nanohydrogels: -unmethylated cytosine-phosphate-guanine oligonucleotides (CpG ODNs) -DNA nanohydrogels (CpG-MUC1-hydrogel) - I-motif cytosine (C)-rich single-stranded DNA	S1(I-motif): TCAACACTAATCCCCAATCCCAATCCCAATCCCAAACG A S2: TCAACACTAATCCGTTTGGGATTGGGACAAAACGACGAA S3(CpG-MUC1): TCAACACTAATCCCCAATCGTCGTTTTGTCGTTTTGTCGTT-S-S GCAGTTGATCCTTTGGATACCCTGG S4: TCAACACTAATCAAAACGACAAAACGATTGGGATTGGGA L1: GATTAGTGTTGAAACGACA-S-S-AAACG L2: ACAAAA-S-S-CGACGAGCCCTCCCCC L3: GATTAGTGTTGAGGGGGAGGGC L4 (CpG): TCGTCGTTTTGTCGTTTTGTCGTT	MCF-7, A549, HepG-2, Female BALB/c (nu/nu) athymic nude mice (5–6 wk)	MUC1	DOX	[28]
antimiR-21 DNAzyme linked to the aptamer of low-density lipoprotein receptor (LDL-R)	TCA ACA GGC TAG CTA CAA CGA CAG TCT GAT AAG CTA TTTTTA GGA CAG GAC CAC ACC CAG CGC GGT CGG CGG GTG GGC GGG GGG AGA ACG AGG TAG GG	Huh-7, MDA-MB-231	LDL-R	antimiR-21 DNAzyme	[29]
Covalently bonding paclitaxel (PTX) to the nucleolin AS1411 aptamer (NucA) through dipeptide bond	TTGGTGGTGGTGGTTGTGGTGGTGGTGG	SKOV3 (ATCC HTB-77), OVCAR3 (ATCC HTB-161)	Nucleolin and cathepsin	PTX	[30]
HER2- aptamer-conjugated to the mertansine (DM1)	AGC CGCGAG GGG AGG GAU AGG GUA GGG CGC GGC U	BT-474, MDA-MB-231 MCF-7, A549, mouse xenografts with BT-474 breast cancer	HER2	DM1	[31]
conjugation of HER2 aptamer to siRNAs targeting Bcl-2	GGGAGGACGAUGCGGCGAUGCUUACGUGCACGCGCCAGACGACUCGCCCGAGCUGUCACAGAGGGGCUACUU	N202.1A	HER2	siRNAs targeting Bcl-2	[32]
^18^F-fluoride- HER2 aptamer	TCCTGGCATGTTCGATGGAGGCCTTTGATTACAGCCCAGA	tumor-bearing mice	HER2	^18^F-fluoride- HER2 aptamer	[33]

**Table 2:** Summarized data of aptasensors for detection of biomarkers.

Method	Sequence	Biomarker	Linear Range	LOD	Ref.
Exosomes on the molecularly imprinted polymer (MIP)-coated Fe_3_O_4_ release the aptamer-FAM from GO	CD63: FAM-CACCCCACCTCGCTCCCGTGACACTAATGCTAMUC1: FAM-CAGCCTGCACTCTAACGCAGTTGATCCTTTGGATAGCCTGGGTTAGA	CD63, MUC1	1.19 × 10^−6^–4.76 × 10^−5^ mol/L	2.27 × 10^−6^ mol/L	[79]
tetrahedral DNA (L1–L4) hybridized with MAB	L1: ACATTCCTAAGTCTGAAACATTACAGCTTGCTACACGAGAAGA GCCGCCATAGTA L2: TCAACTGCCTGGTGATAAAACGACACTACGTGGGAATCTACTA TGGCGGCTCTTCTTTTTAATCCGTCGAGCAGAGTT L3: TATCACCAGGCAGTTGACAGTGTAGCAAGCTGTAATAGATGCG AGGGTCCAATACTTTTT/iBHQ2dT/TGCACCCTAACCCTAACCCT L4: TTCAGACTTAGGAATGTGCTTCCCACGTAGTGTCGTTTGTATTG GACCCTCGCAT MB: Cy3-AACGATTAGGGTTAGGGTCGTT-Cy5	telomerase	50–2000 HeLa cells	35 HeLa cells	[80]
Prevention of AuNP aggregation by aptamer	CCGATCTCTCCCACTCTCTCCAACTCACAGGCTACGGCACGTAGAGCATCACCATGATCCTGTGGGTGTGTTGTTGATGGATCGGATCATCATGGTGAT	platelet-derived growth factor (PDGF)	0.01–10 μg/mL	0.01 μg/mL	[81]
Gold chip modified with CA125 aptamer through streptavidin–biotin	CTC ACT ATA GGG AGA CAA GAA TAA ACG CTC AA-biotin	Mucin 16 (MUC16) or cancer antigen 125 (CA125)	10–100 U/mL	0.01 U/mL	[82]
Magnetic glass carbon electrode (MGCE)/α-Fe_2_O_3_/Fe_3_O_4_/Au/complementary strand/aptamer	Complementary strand: SH-TTTTTTTTTTTTTTTTTTTTCCCTATAGTGAG Aptamer: CTCACTATAGGGAGACAAGAATAAACGCTCAA	cancer antigen 125 (CA125)	5–125 U/mL	2.99 U/mL	[83]
CRISPR/Cas12a + RPA + electrochemistry (Modified electrode with complementary 1 (CP1) and MB/Fe_3_O_4_@COF/PdAu modified with complementary 2 (CP2))	crRNA template: AAGTACCCAGCAGTTTGGCCCGCCATCTACACTTAGTAGAAATTCCtatagtgagtcgtattag CP1: SH-ACACTTGAAGTGTATTTCCTAAATA CP2: AATTGCAAGTATGTAGAAGTTCACA-SH Synthetic target: ATGTCAAGATCACAGATTTTGGGCTGGCCAAACTGCTGGGTGCG	ctDNA EGFR L858R	10 aM–100 pM	3.3 aM	[84]
Microfluidic system incorporated with screen-printed gold electrode was modified with integrin α6β4-specific aptamer (IDA)	GCCTGTTGTGAGCCTCCTAACCGTGCGTATTCGTACTGGAACTGATATCGATGTCCCCATGCTTATTCTTGTCTCCC–SH	α6β4 integrin on A549 cells	50–5 × 10^5^ cells/mL	14 cells/mL	[85]
Lateral flow assay (LFA) with streptavidin (SA)-biotin-CD63 aptamer on T-line	CD63 aptamer: 5′-GTGGGGTGGACGAGGGCACGTGATTACGTA-3′ complement aptamer: CACCCCACCTCGCTCCCGTGACACTAATGCTA -Biotin	CD63 on the non-small cell lung cancer (NSCLC)	-	6.4 × 10^9^ particles/mL	[86]
Smartphone control of emission from gold nanocluster (GNC)-aptamer as emitter and polyurethane (PU) coated with GO as quencher	SH-CCCCCCGATCCTTTGGATA	Mucin 1 (MUC1)	250–20,000 cells/mL	221 cells/mL	[87]

## Data Availability

Not applicable.
